# Correlation between triglyceride glucose index and collateral circulation formation in patients with chronic total occlusion of coronary arteries in different glucose metabolic states

**DOI:** 10.1186/s12933-023-02080-3

**Published:** 2024-01-13

**Authors:** Junwei Zhu, Minghui Niu, Chenxing Wang, Ying Liang, Rong Guo, Fei He

**Affiliations:** 1 Department of Cardiology, The First Affiliated Hospital of Zhengzhou University, Zhengzhou, China; 2 Department of Hematology, The First Affiliated Hospital of Zhengzhou University, Zhengzhou, China

**Keywords:** Chronic total occlusion, Coronary collateral circulation, Triglyceride glucose index, Different glucose metabolic states

## Abstract

**Background:**

To investigate the correlation between triglyceride glucose index (TyG) and collateral circulation in patients with chronic total occlusion (CTO) of coronary arteries in different glucose metabolic states.

**Methods:**

A total of 681 patients who underwent coronary angiography between January 2020 and December 2021 to determine the presence of CTO lesions in at least one major coronary artery were retrospectively included in this study. Patients were categorized into a group with poor collateral circulation formation (Rentrop grade 0–1, *n* = 205) and a group with good collateral circulation formation (Rentrop grade 2–3, *n* = 476) according to the Rentrop scale. They were also categorized according to their glucose metabolism status: normal glucose regulation (NGR) (*n* = 139), prediabetes mellitus (Pre-DM) (*n* = 218), and diabetes mellitus (DM) (*n* = 324). Correlation between TyG index and collateral circulation formation was analyzed by logistic regression analysis and receiver operating characteristic (ROC) curves.

**Results:**

Among patients with CTO, TyG index was significantly higher in the group with poor collateral circulation formation than in the group with good collateral circulation formation. Logistic regression analysis showed that TyG index was an independent risk factor for poor collateral circulation formation (OR 5.104, 95% CI 3.323–7.839, *P* < 0.001). The accuracy of TyG index in predicting collateral circulation formation was evaluated by the ROC curve, which had an area under the curve of 0.779 (95% CI 0.738–0.820, *P* < 0.001). The restrictive cubic spline curves showed that the risk of poor collateral circulation formation in the Pre-DM and DM groups was initially flat and finally increased rapidly, except for the NGR group. TyG index was significantly associated with an increased risk of poor collateral circulation formation in the Pre-DM and DM groups.

**Conclusions:**

TyG index was significantly associated with the risk of poor collateral circulation formation in patients with CTO, especially those with Pre-DM and DM.

**Supplementary Information:**

The online version contains supplementary material available at 10.1186/s12933-023-02080-3.

## Introduction

Chronic total occlusion (CTO) of the coronary arteries is defined as a lesion in which a coronary artery is completely occluded for at least 3 months [1]. One study [2] reported that the prevalence of CTO in patients who underwent coronary angiography (CAG) to confirm the diagnosis of coronary artery disease was 20%. Percutaneous coronary intervention (PCI) in patients with CTO has a low success rate, a high risk of procedural complications, a long and costly procedure time, and unclear clinical benefits relative to patients without CTO [3, 4]. Previous studies have shown [5–7] that good coronary collateral circulation (CCC) reduces infarct size after acute myocardial infarction, decreases the risk of post-infarction complications, reduces the number of angina episodes, and decreases cardiovascular and all-cause mortality. Although the severity of coronary artery lesions is similar in different CTO patients, the severity of the disease is not identical, which may be related to the degree of CCC formation [8]. Currently, assessment of CCC formation in patients with CTO relies on invasive CAG, noninvasive assessment methods are complex and expensive to perform, and there is a lack of simple and easy predictive assessment indexes [9]. Therefore, there is a need to find a simple and effective biomarker to assess or predict CCC formation.

Insulin resistance (IR) has been shown to be an independent risk factor for poor collateral circulation formation [10]. Insulin resistance can be assessed by a variety of metrics, such as fasting insulin levels, normoglycemic clamp method, and homeostasis model assessment-IR (HOMA-IR) in vivo [11–13], however, these metrics are not routinely measured in clinical practice, especially in nondiabetic patients. The triglyceride glucose (TyG) index, calculated from the combination of fasting glucose and triglycerides, has been recognized as a novel biomarker of insulin resistance [14]. Wu et al. [15] confirmed that TyG index can predict the occurrence of early-onset coronary heart disease adverse cardiovascular events. Furthermore, a meta-analysis [16] showed that TyG index is significantly associated with the risk of coronary artery disease and stroke. A high TyG index is also positively associated with carotid plaque load in individuals with prediabetes (Pre-DM) and new-onset type 2 diabetes mellitus (DM) [17]. Meanwhile, Liu et al. [18] found that TyG index is associated with arterial stiffness and coronary artery calcification. Based on these observations, it is hypothesized that as a novel marker for assessing insulin resistance, TyG index may be associated with CCC formation in patients with CTO. Currently, there are limited studies on the correlation between TyG index and CCC formation in patients with CTO. Gao et al. [19] initially investigated the correlation between TyG index and collateral circulation in patients with CTO, but no studies have explored the correlation between TyG index and collateral circulation in CTO patients under different glucose metabolic states, even though TyG index is closely related to glucose metabolism. In this study, we aimed to investigate, for the first time, the correlation between TyG index and collateral circulation in CTO patients at different glucose metabolic states.

## Methods

### Study design and population

**Fig. 1:**
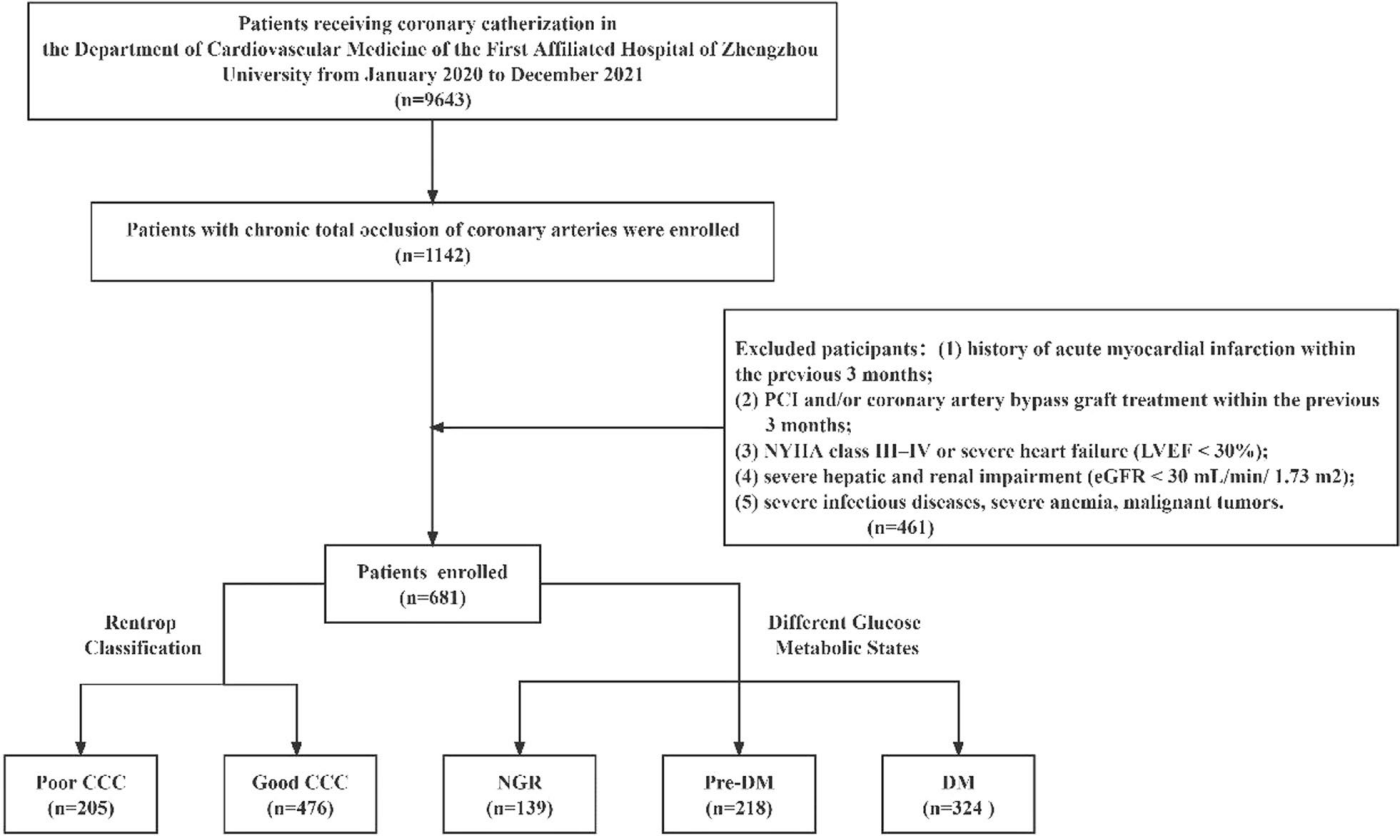
Flow chart of patient recruitment. *CCC* coronary collateral circulation, *NGR* normal glucose regulation, *Pre-DM* prediabetes mellitus, *DM* diabetes mellitus

Study participants included 681 patients who were hospitalized in the Department of Cardiovascular Medicine of the First Affiliated Hospital of Zhengzhou University from January 2020 to December 2021 and underwent coronary angiography (CAG), with at least one major epicardial coronary artery CTO lesion identified by angiographic results (Fig. 1). The study protocol was approved by the Ethics Committee of the First Affiliated Hospital of Zhengzhou University, and written informed consent was obtained from all participants.

The diagnosis of CTO lesions was based on the diagnostic criteria developed by the American Heart Association in 2011, that is, in the coronary arteries on the basis of atherosclerotic lesions due to thrombus formation, recurrent mechanization leading to complete obstruction of coronary vascular lumen and the duration of the occlusion was more than 3 month [20].

Exclusion criteria were (1) history of acute myocardial infarction within the previous 3 months, (2) PCI and/or coronary artery bypass graft treatment within the previous 3 months, (3) NYHA class III–IV or severe heart failure (left ventricular ejection fraction [LVEF] < 30%), (4) severe hepatic and renal impairment (estimated glomerular filtration rate [eGFR] < 30 mL/min/1.73 m^2^), and (5) severe infectious diseases, severe anemia, malignant tumors, etc.

### Data collection and definitions

For cases meeting the inclusion criteria, personal information for each patient such as name, gender, age, smoking history, drinking history, etc., as well as LVEF, systolic blood pressure, and diastolic blood pressure measured in the right upper arm were collected by medical professionals at the time of admission. Venous blood specimens were collected by medical professionals early in the morning on the day after admission following fasting for at least 8 h. The blood samples were analyzed by fully automated hematology analyzers to obtain measurements for total cholesterol (TC), low-density lipoprotein cholesterol (LDL-C), high-density lipoprotein cholesterol (HDL-C), triglycerides (TG), fasting plasma glucose (FPG), glycosylated hemoglobin A1c (HbA1c), C-reactive protein (CRP), and creatinine (Cr). All patients underwent CAG by the radial or femoral artery route using the standard Judkins method, and the results were described and recorded by two senior interventional cardiologists.

TyG index was calculated according to the following formula: TyG index = Ln [TG (mg/dL) × FPG (mg/dL)/2] [14]. Coronary artery disease was defined as ≥ 50% luminal narrowing of at least one coronary artery (left anterior descending, left circumflex, or right coronary arteries). According to the American Diabetes Association's Standards for the Medical Management of Diabetes, DM was defined as FPG ≥ 7.0 mmol/L or HbA1c ≥ 6.5%, Pre-DM was defined as 5.6 mmol/L ≤ FPG ≤ 6.9 mmol/L or 5.7% ≤ HbA1c ≤ 6.4%, and normal glucose regulation (NGR) was defined as FPG < 5.6 mmol/L or HbA1c < 5.7% [21]. The Rentrop classification was used to evaluate collateral circulation and included four grades: grade 0, no collateral vessels were filled with contrast, grade 1, collateral vessels were filled with contrast but did not perfuse the epicardial arteries, grade 2, the epicardial arteries were partially filled with contrast through the collateral vessels, and grade 3, the epicardial arteries were completely filled with contrast through the collateral vessels. In patients with multiple coronary lesions, the side branch with the highest Rentrop classification was used when there were multiple coronary side branches [22]. According to the Rentrop grading, patients were divided into two groups: one with poor collateral circulation formation (Rentrop grade 0–1) and one with good collateral circulation formation (Rentrop grade 2–3).

### Statistical analysis

The Kolmogorov–Smirnov test was used to assess the normality of the measurement data, which was expressed as mean ± standard deviation for normal distribution, median and interquartile spacing for non-normal distribution, and percentage for categorical variables. When grouped by the formation of collateral circulation, the t test or Mann–Whitney U test were used to compare continuous variables between the two groups, when grouped by different glucose metabolic states, analysis of variance or Kruskal–Wallis test were used to compare continuous variables between the three groups. Categorical variables were compared by χ2 test. Multicollinearity was tested in multivariable models with a variance inflation factor threshold of < 5. We found multicollinearity between TG, FPG, and TyG index. Using univariate logistic regression analysis, we found that TC, HDL-C, HbA1c, and TyG index were statistically significant (*P* < 0.05), and we included them in a multivariable logistic regression analysis to calculate odds ratios (OR) and 95% confidence intervals (CI) in order to test for a correlation between TyG index and collateral circulation in patients with CTO. Restricted cubic spline analysis was performed to reflect the dose–response relationship between TyG index and the risk of poor collateral circulation formation in different glucose metabolic states. The sensitivity and specificity of the TyG index in predicting the formation of collateral circulation were evaluated using the subjects' work characteristic curve (ROC) and area under the curve (AUC). All data were analyzed using R version 4.1.0, GraphPad Prism version 8.0.1, and SPSS for windows version 25. A P-value of < 0.05 was considered statistically significant.

## Results

### Clinical baseline data grouped according to collateral circulation

Study participants were grouped based on the formation of collateral circulation, which resulted in 205 cases in the poor collateral circulation group and 476 cases in the good collateral circulation group. TyG index, FPG, HbA1c, TC, and TG were significantly higher in the poor CCC formation group compared with the good CCC group (*P* < 0.001), whereas HDL-C showed the opposite trend, being significantly lower in the poor CCC formation group than in the good CCC group (*P* = 0.026). The proportion of patients with DM and Pre-DM was significantly higher in the poor CCC group compared with the good CCC group (*P* < 0.001). In terms of age and sex ratio, there was no difference between the two groups (*P* > 0.05) (Additional file 1: Table S1).

### Multifactorial analysis of factors related to the formation of collateral circulation

Multifactorial logistic regression analysis was performed using good or poor formation of collateral circulation as the dependent variable, and each factor that was statistically significant (*P* < 0.05) in the one-way analysis as the independent variable. The results of this analysis showed that TyG index (OR 5.104, 95% CI 3.323–7.839, *P* < 0.001) and HbA1c (OR 1.278, 95% CI 1.120–1.458, *P* < 0.001) were independent correlates affecting the formation of CCC (Additional file 1: Table S2).

### Baseline data of different glucose metabolism status groupings

When the patients with CTO were grouped according to glucose metabolism status, there were 139 cases in the NGR group, 218 cases in the Pre-DM group, and 324 cases in the DM group. There were significant differences between the three groups in HDL-C, TG, TyG index, FPG, and HbA1c (*P* < 0.05 for all), and the percentage of poor collateral circulation formation was significantly higher in the Pre-DM and DM groups compared with that in the NGR group (*P* < 0.001 for all) (Table 1). HDL-C in the DM group was significantly lower than that in the NGR group (*P* = 0.0075), but was not significantly different from that in the Pre-DM group (*P* = 0.7769), and HDL-C in the Pre-DM group was not significantly different from that in the NGR group (*P* = 0.1674) (Additional file 1: Fig. S1 a). TG in the DM group was significantly higher than that in the NGR group and the Pre-DM group (both *P* < 0.05), and TG in the Pre-DM group was not significantly different from that of the NGR group (*P* = 0.2345) (Additional file 1: Fig. S1 b).TG, TyG index, FPG, and Hb1Ac in the DM group were significantly higher than those in the NGR group and the Pre-DM group (all *P* < 0.05), TG in the Pre-DM group was not significantly different from that in the NGR group (*P* = 0.2345), and TyG index, FPG, and Hb1Ac in the Pre-DM group were significantly higher than those in the NGR group (all *P* < 0.05) (Additional file 1: Fig. S1b–e).

**Table 1:** Clinical baseline data grouped according to different glucose metabolic status

	NGR	Pre-DM	DM	*P*
	(n = 139)	(n = 218)	(n = 324)	
Age (years)	59.37 ± 9.49	59.57 ± 9.86	60.06 ± 10.42	0.747
Male (n, %)	116 (83.5%)	171 (78.4%)	243 (75.0%)	0.129
SBP (mmHg)	134.06 ± 17.77	133.67 ± 18.88	133.09 ± 16.73	0.106
DBP (mmHg)	79.68 ± 12.52	78.23 ± 11.72	78.46 ± 10.45	0.101
Smoking history (n, %)	55 (39.6%)	55 (43.6%)	135 (41.7%)	0.752
Drinking history (n, %)	53 (23.3%)	63 (27.8%)	59 (26.0%)	0.731
History of hypertension (n, %)	84 (60.4%)	120 (55.0%)	198 (61.1%)	0.346
Previous medication				
Antihypertensive drugs (n, %)	83 (59.7%)	119 (54.6%)	191 (59.0%)	0.521
Lipid-lowering drugs (n, %)	133 (95.7%)	205 (94.0%)	310 (95.7%)	0.648
Antiplatelet drugs (n, %)	129 (92.8%)	201 (92.2%)	300 (92.6%)	0.975
Antidiabetic drugs (n, %)	9 (6.5%)	27 (12.4%)	166 (51.2%)	< 0.001*
Laboratory examination				
cTnI (ng/mL)	0.010 (0.010–0.020)	0.010 0.010–0.020)	0.010 (0.010–0.020)	0.425
BNP (pg/mL)	131.79 ± 61.11	128.03 ± 56.08	130.85 ± 59.81	0.821
Cr (μmol/L)	70.16 ± 12.83	70.23 ± 13.68	68.47 ± 14.53	0.592
eGFR (mL/min/1.73 m^2^)	92.03 ± 12.19	93.28 ± 12.67	68.47 ± 14.53	0.470
CRP (mg/L)	1.26 (0.74–1.83)	1.34(0.77–1.90)	1.32 (0.78–1.83)	0.668
TC (mg/dL)	3.36 (2.88–3.91)	3.58(3.00–4.29)	3.54 (3.09–4.22)	0.083
TG (mg/dL)	1.22 (0.90–1.75)	1.38(1.03–1.81)	1.56 (1.10–2.11)	< 0.001*
HDL-C (mg/dL)	0.96 (0.82–1.15)	0.91 (0.79–1.05)	0.90 (0.78–1.04)	0.010*
LDL-C (mg/dL)	1.97 (1.57–2.42)	2.07 (1.65–2.64)	2.10 (1.66–2.61)	0.118
FPG (mmol/L)	4.60 (4.25–5.05)	5.01 (4.57–5.46)	7.20 (6.01–9.05)	< 0.001*
HbA1c (%)	5.40 (5.30–5.50)	6.00 (5.80–6.20)	7.60 (6.90–9.00)	< 0.001*
LVEF (%)	61.00 (57.50–64.00)	61.00 (52.00–63.00)	61.00 (52.00–64.00)	0.312
TyG index	8.43 (8.06–8.81)	8.63 (8.37–8.89)	9.11 (8.70–9.52)	< 0.001*
Number of vascular stenosis				
1	17 (12.2%)	21 (9.6%)	24 (7.4%)	0.580
2	33 (23.7%)	52 (23.9%)	81 (25%)	
3	89 (64.1%)	145 (66.5%)	219 (67.6%)	
CTO related artery				
LAD	64 (38.1%)	95 (34.3%)	138 (34.5%)	0.784
LCX	36 (21.4%)	69 (24.9%)	105 (26.3%)	
RCA	68 (40.5%)	113 (40.8%)	157 (39.3%)	
Rentrop collateral grading				
0	5 (3.6%)	11 (5.0%)	38 (11.7%)	< 0.001*
1	19 (13.7%)	31 (14.2%)	101 (31.2%)	
2	47 (33.8%)	86 (39.4%)	121 (37.3%)	
3	68 (48.9%)	90 (41.3%)	64 (19.8%)	

*SBP* systolic blood pressure, *DBP* diastolic blood pressure, *cTnI* cardiac troponin I, *NT-pro BNP* N-terminal B-type natriuretic peptide, *Cr* creatinine, *eGFR* estimated glomerular filtration rate, *CRP* C-reactive protein, *TC* total cholesterol, *TG* triglyceride, *HDL-C* high-density lipoprotein cholesterol, *LDL-C* low-density lipoprotein cholesterol, *FPG* fasting plasma glucose, *HbA1c* glycated hemoglobin, *LVEF* left ventricular ejection fraction, *TyG* triglyceride glucose, *NGR* normal glucose regulation, *Pre-DM* prediabetes mellitus, *DM* diabetes mellitus, *LAD* left anterior descending artery, *LCX* left circumflex coronary artery, *RCA* right coronary artery

*Statistically significant difference between two groups

### Relationship between TyG index and collateral circulation in different glucose metabolic states

Participants were divided into NGR group, Pre DM group, and DM group based on their glucose metabolism status, with the formation of collateral circulation as the dependent variable and statistically significant (P < 0.05) factors in univariate analysis as independent variables. The multivariable logistic regression analysis was performed by substituting them into the multivariable analysis equation. In the NGR group, we found no correlation between TyG index and poor CCC formation (P > 0.05). In the Pre-DM group, TyG index (OR 6.487, 95% CI 2.460–17.101, P < 0.001) was found to be an independent correlate affecting CCC formation. In the DM group, TyG index (OR 6.692, 95% CI 3.648–12.157, P < 0.001) and HbA1c (OR 1.371, 95% CI 1.125–1.671, P < 0.001) were independent correlates affecting the formation of CCC (Table 2). The restricted cubic spline curve showed that the risk of poor collateral circulation formation in Pre-DM and DM groups was initially flat and then increased rapidly, except for the NGR group (Additional file 1: Fig. S2) (Table 3)

**Table 2:** Correlations between TyG index and poor Coronary Collateral Circulation in different glucose metabolism states

		Univariate analysis			Multivariate analysis		
		OR (95% CI)	β	*P*	OR (95% CI)	β	*P*
NGR	TyG index	1.733 (0.770–3.900)	0.55	0.184			
Pre-DM	TC	1.414 (1.020–1.959)	0.346	0.038	1.160 (0.783–1.717)	0.148	0.459
	HDL-C	0.120 (0.019–0.774)	− 2.120	0.026	0.363 (0.042–3.104)	− 1.014	0.354
	TyG index	8.224 (3.459–19.557)	2.107	< 0.001	6.487 (2.460–17.101)	1.870	< 0.001
DM	TC	1.605 (1.230–2.094)	0.473	< 0.001	1.138 (0.815–1.588)	0.129	0.448
	HDL-C	0.285 (0.090–0.902)	− 1.257	0.033	0.791 (0.181–3.453)	− 0.235	0.791
	HbA1c	1.716 (1.437–2.048)	0.540	< 0.001	1.371 (1.125–1.671)	0.315	0.002
	TyG index	9.283 (5.341–16.136)	2.228	< 0.001	6.692 (3.648–12.157)	1.901	< 0.001

*OR* odds ratios, *CI* confidence interval, *NGR* normal glucose regulation, *Pre-DM* prediabetes mellitus, *DM* diabetes mellitus, *TC* total cholesterol, *HDL-C* high-density lipoprotein cholesterol, *HbA1c* glycosylated hemoglobin A1c, *TyG* triglyceride glucose

*Pre-DM* TC was adjusted for HDL-C, *TyG index* TyG index was adjusted for TC, HDL-C HDL-C was adjusted for TC, TyG index, *DM* TC was adjusted for HDL-C, HbA1c, TyG index HDL-C was adjusted for TC, HbA1c, TyG index HbA1c was adjusted for TC, HDL-C, TyG index was adjusted for TC, HDL-C, HbA1c

**Table 3:** The predictive value of TyG index for poor coronary collateral circulation

	Variable	AUC	95% CI	Cutoff point	Sensitivity	Specificity	*P*
Total	TyG index	0.799	0.738–0.820	9.155	0.624	0.874	< 0.001
NGR	TyG index	0.565	0.441–0.690	7.955	0.985	0.199	0.314
Pre-DM	TyG index	0.739	0.633–0.845	9.150	0.476	0.943	< 0.001
DM	TyG index	0.801	0.751–0.851	9.215	0.712	0.811	< 0.001

*AUC* area under the curve, *CI* confidence interval, *NGR* normal glucose regulation, *Pre-DM* prediabetes mellitus, *DM* diabetes mellitus

### Predictive value of TyG index for poor collateral circulation formation in patients with CTO

We compared the AUC of TyG index with TC, TG, HDL-C and HbA1c in different glucose metabolic states. The results showed that in the NGR group, the AUC of TyG index was not significantly different from TC, TG, HDL-C and HbA1c. However, in the Pre-DM, DM, and baseline groups, we observed statistically significant differences between the AUC of the TyG index and the AUC of TC, TG, HDL-C, and HbA1c (*P* < 0.05). These results suggest that TyG index is superior to TC, TG, HDL-C and HbA1c in predicting poor collateral circulation formation (Additional file 1: Table S3, Fig. S3).

**Fig. 2:**
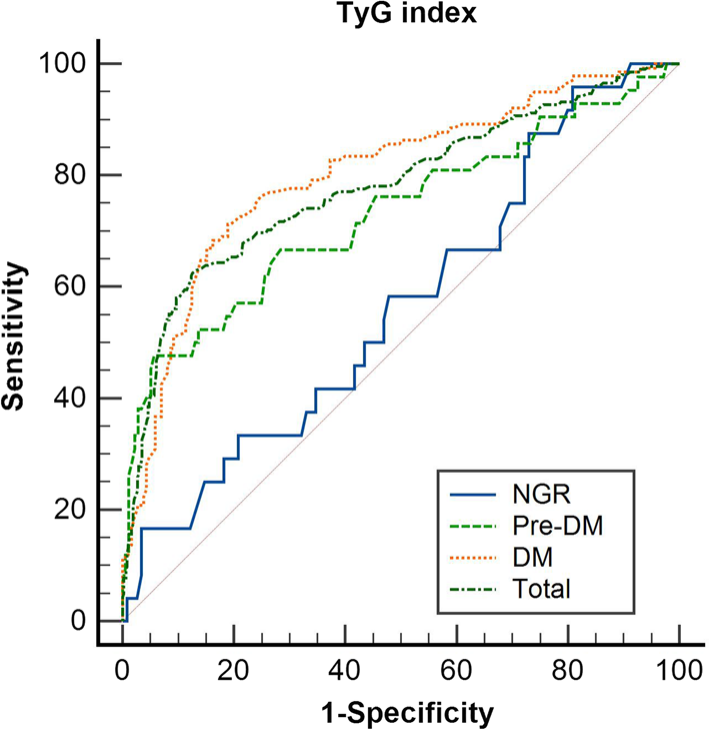
The TyG index predicts that poor coronary collateral circulation ROC curve. *NGR* normal glucose regulation, *Pre-DM* prediabetes mellitus, *DM* diabetes mellitus

The ROC curve for poor coronary collateral circulation and TyG index is shown in Fig. 2. When TyG index was added to the baseline model, the optimal cutoff for predicting poor collateral circulation formation was 9.155. At this cutoff value, the sensitivity was 62.4%, the specificity was 87.4%, and the AUC was 0.799 (95% CI 0.738–0.820, *P* < 0.001). When TyG index was added to the NGR model, the result showed that TyG index was not significant in predicting poor collateral circulation formation (*P* = 0.314). When TyG index was added to the Pre-DM model, the optimal cutoff for predicting poor collateral circulation formation was 9.150. At this cutoff value, the sensitivity was 47.6%, the specificity was 94.3%, and the AUC was 0.739 (95% CI 0.633–0.845, *P* < 0.001). When TyG index was added to the DM model, the optimal cutoff value for predicting poor collateral circulation formation was 9.215. At this cutoff value, the sensitivity was 7.12%, the specificity was 81.1%, and the AUC was 0.801 (95% CI 0.751–0.851, *P* < 0.001) (Table 3). There was no statistically significant difference in AUC of TyG index between the baseline model, Pre-DM model and DM model (*P* > 0.05) (Additional file 1: Table S4).

## Discussion

The present study showed that TyG index was significantly associated with the risk of poor collateral circulation formation, especially in the pre-DM and DM groups. Importantly, this is the first study to reveal the correlation between TyG index and the risk of poor collateral circulation formation in different glucose metabolic states.

The main advantage of the TyG index is that it is calculated from fasting glucose and triglycerides and does not require the measurement of serum insulin, thus TyG index is a simple and easy-to-use technique for evaluating insulin resistance [23–25]. Several studies have reported that cardiovascular events are closely related to the TyG index. Sanchez-Inigo et al. [26] found that the TyG index can predict the occurrence of adverse cardiovascular events, and Luo et al. [27] found that a higher TyG index is associated with an increased risk of adverse cardiovascular events after PCI for acute ST-segment elevation myocardial infarction. Guo et al. [28] suggested that the TyG index is a good indicator for the occurrence of adverse cardiovascular events in prediabetic patients. TyG index is widely used in the study of cardiovascular diseases.

In this study, TyG index was found to be an independent risk factor for poor collateral circulation formation, which was consistent with the findings of Gao [19] et al. Our ROC curve analysis showed that TyG index predicted poor collateral circulation formation with an AUC of 0.799, a sensitivity of 62.4%, and a specificity of 87.4%. This suggests that TyG index could be used as a simple, easy and inexpensive noninvasive biomarker to predict and evaluate good CCC formation in CTO patients in daily clinical practice.

The effect of the TyG index on collateral circulation formation suggests that insulin resistance plays a crucial role in the formation of collateral circulation in patients with CTO. Insulin resistance causing compensatory hyperinsulinemia can impair the insulin signaling pathway in vascular endothelial cells, leading to decreased nitric oxide (NO) production and vasodilatory dysfunction, which in turn causes vascular endothelial dysfunction [29–31]. Hyperinsulinemia can cause impaired expression of vascular endothelial growth factor in the heart [32]. In addition, insulin resistance can lead to disturbances in glucose metabolism and produce chronic hyperglycemia. Chronic hyperglycemia can cause an increase in free radicals through different pathways, which in turn triggers oxidative stress leading to cellular damage [33–35]. Furthermore, insulin resistance can cause an elevation of free fatty acids (FFA) in the blood, which can lead to mitochondrial dysfunction and an increase in reactive oxygen species production. FFA can also lead to the activation of inflammatory factors (TNF-α, IL1-β, and IL-6) and an elevation of monocyte chemotactic protein-1 (MCP-1), which can cause cellular damage and chronic inflammation [36]. All these factors may hamper angiogenesis and arteriogenesis, thereby inhibiting the formation of collateral circulation [37].

In this study, patients with CTO were also divided into NGR, Pre-DM, and DM groups according to their glucose metabolism status, and it was found that TyG index was not correlated with poor collateral circulation formation in the NGR group but was significantly correlated with the risk of poor collateral circulation formation in CTO patients in the Pre-DM group and the DM group. Furthermore, TyG index was an independent risk factor for poor collateral circulation formation in the latter two groups This suggests that the relationship between TyG index and collateral circulation formation differs for different glucose metabolic states. We hypothesize that this mechanism may be due to the fact that insulin resistance is more severe in Pre-DM, and DM patients than in those with NGR. Previous studies have shown that patients with Pre-DM have higher levels of insulin resistance compared to those with NGR [38]. Insulin resistance is an important pathophysiologic pathway that contributes to the development of diabetes and may be present for an extended period of time before the diagnosis of diabetes is made [39, 40].

In conclusion, this study found for the first time that TyG index was significantly associated with the risk of poor collateral circulation formation in patients with CTO, especially those with Pre-DM and DM. There are several limitations of this study that are worth considering. First, this is a single-center retrospective study with a limited sample size. Second, the results of this study were only for the Chinese population, so caution must be exercised in generalizing the results to other populations because a causal relationship could not be established. Third, the study did not differentiate between diabetic patients with well-controlled disease and diabetic patients with uncontrolled disease. Fourth, survival bias due to fatal events should not be overlooked. Fifth, there is a lack of information about glucocorticosteroids and fenofibrate drugs that may affect serum TG levels. In addition, possible survival bias due to fatal events should be recognized. Finally, insulin is not a common laboratory parameter in patients with CAD, especially in nondiabetic patients, and therefore no comparison of HOMA-IR and TyG index was performed.

### Supplementary Information

**Additional file 1: Table S1.** Clinical baseline information according to the grouping of collateral circulation. **Table S2.** Poor collateral circulation formation in relation to various risk factors. **Table S3.** Comparison of TyG index and other factors AUC in different glucose metabolic states. **Table S4.** Comparison of AUC of TyG index in different glucose metabolic states. **Figure S1.** Biochemical indexes in different glucose metabolic states. **a** HDL-C, **b** TG, **c** TyG index, **d** FPG, **e** HbA1c. **Figure S2.** TyG index and poor CCC restricted cubic spline curves in different glucose metabolic states. **a** Normal glucose regulation, **b** prediabetes mellitus, **c** diabetes mellitus. **Figure S3.** TyG index and other factors predicted poor collateral circulation formation in different glucose metabolic states.

## Data Availability

The raw data supporting the conclusions of this article will be made available by the authors, without undue reservation.

## Abbreviations

SBP: Systolic blood pressure
DBP: Diastolic blood pressure
cTnI: Cardiac troponin I
NT-pro BNP: N-terminal B-type natriuretic peptide
Cr: Creatinine
eGFR: Estimated glomerular filtration rate
CRP: C-reactive protein
TC: Total cholesterol
TG: Triglyceride
HDL-C: High-density lipoprotein cholesterol
LDL-C: Low-density lipoprotein cholesterol
FPG: Fasting plasma glucose
HbA1c: Glycated hemoglobin
LVEF: Left ventricular ejection fraction
TyG: Triglyceride glucose
NGR: Normal glucose regulation
Pre-DM: Prediabetes mellitus
DM: Diabetes mellitus
LAD: Left anterior descending artery
LCX: Left circumflex coronary artery
RCA: Right coronary artery
*: Statistically significant difference between two groups

## References

[1] Stone GW, Kandzari DE, Mehran R, Colombo A, Schwartz RS, Bailey S, Moussa I, Teirstein PS, Dangas G, Baim DS (2005). Percutaneous recanalization of chronically occluded coronary arteries: a consensus document: part I. Circulation.

[2] Azzalini L, Jolicoeur EM, Pighi M, Millan X, Picard F, Tadros VX, Fortier A, L'Allier PL, Ly HQ (2016). Epidemiology, management strategies, and outcomes of patients with chronic total coronary occlusion. Am J Cardiol.

[3] Othman H, Seth M, Zein R, Rosman H, Lalonde T, Yamasaki H, Alaswad K, Menees D, Mehta RH, Gurm H (2020). Percutaneous coronary intervention for chronic total occlusion—the Michigan experience: insights from the BMC2 registry. JACC Cardiovasc Interv.

[4] Shah PB (2011). Management of coronary chronic total occlusion. Circulation.

[5] Seiler C, Stoller M, Pitt B, Meier P (2013). The human coronary collateral circulation: development and clinical importance. Eur Heart J.

[6] Berry C, Balachandran KP, L'Allier PL, Lesperance J, Bonan R, Oldroyd KG (2007). Importance of collateral circulation in coronary heart disease. Eur Heart J.

[7] Charney R, Cohen M (1993). The role of the coronary collateral circulation in limiting myocardial ischemia and infarct size. Am Heart J.

[8] Meier P, Hemingway H, Lansky AJ, Knapp G, Pitt B, Seiler C (2012). The impact of the coronary collateral circulation on mortality: a meta-analysis. Eur Heart J.

[9] Traupe T, Gloekler S, de Marchi SF, Werner GS, Seiler C (2010). Assessment of the human coronary collateral circulation. Circulation.

[10] Mouquet F, Cuilleret F, Susen S, Sautiere K, Marboeuf P, Ennezat PV, McFadden E, Pigny P, Richard F, Hennache B (2009). Metabolic syndrome and collateral vessel formation in patients with documented occluded coronary arteries: association with hyperglycaemia, insulin-resistance, adiponectin and plasminogen activator inhibitor-1. Eur Heart J.

[11] Antuna-Puente B, Disse E, Rabasa-Lhoret R, Laville M, Capeau J, Bastard JP (2011). How can we measure insulin sensitivity/resistance?. Diabetes Metab.

[12] Okita K, Iwahashi H, Kozawa J, Okauchi Y, Funahashi T, Imagawa A, Shimomura I (2013). Homeostasis model assessment of insulin resistance for evaluating insulin sensitivity in patients with type 2 diabetes on insulin therapy. Endocr J.

[13] Wilcox G (2005). Insulin and insulin resistance. Clin Biochem Rev.

[14] Simental-Mendía LE, Rodríguez-Morán M, Guerrero-Romero F (2008). The product of fasting glucose and triglycerides as surrogate for identifying insulin resistance in apparently healthy subjects. Metab Syndr Relat Disord.

[15] Wu Z, Liu L, Wang W, Cui H, Zhang Y, Xu J, Zhang W, Zheng T, Yang J (2022). Triglyceride-glucose index in the prediction of adverse cardiovascular events in patients with premature coronary artery disease: a retrospective cohort study. Cardiovasc Diabetol.

[16] Ding X, Wang X, Wu J, Zhang M, Cui M (2021). Triglyceride-glucose index and the incidence of atherosclerotic cardiovascular diseases: a meta-analysis of cohort studies. Cardiovasc Diabetol.

[17] Jiang ZZ, Zhu JB, Shen HL, Zhao SS, Tang YY, Tang SQ, Liu XT, Jiang TA (2022). A high triglyceride-glucose index value is associated with an increased risk of carotid plaque burden in subjects with prediabetes and new-onset type 2 diabetes: a real-world study. Front Cardiovasc Med.

[18] Liu F, Ling Q, Xie S, Xu Y, Liu M, Hu Q, Ma J, Yan Z, Gao Y, Zhao Y (2023). Association between triglyceride glucose index and arterial stiffness and coronary artery calcification: a systematic review and exposure-effect meta-analysis. Cardiovasc Diabetol.

[19] Gao A, Liu J, Hu C, Liu Y, Zhu Y, Han H, Zhou Y, Zhao Y (2021). Association between the triglyceride glucose index and coronary collateralization in coronary artery disease patients with chronic total occlusion lesions. Lipids Health Dis.

[20] Levine GN, Bates ER, Blankenship JC, Bailey SR, Bittl JA, Cercek B, Chambers CE, Ellis SG, Guyton RA, Hollenberg SM (2011). 2011 ACCF/AHA/SCAI guideline for percutaneous coronary intervention: a report of the American college of cardiology foundation/American heart association task force on practice guidelines and the society for cardiovascular angiography and interventions. Circulation.

[21] American Diabetes A (2014). Diagnosis and classification of diabetes mellitus. Diabetes Care.

[22] Rentrop KP, Cohen M, Blanke H, Phillips RA (1985). Changes in collateral channel filling immediately after controlled coronary artery occlusion by an angioplasty balloon in human subjects. J Am Coll Cardiol.

[23] Guerrero-Romero F, Simental-Mendía LE, González-Ortiz M, Martínez-Abundis E, Ramos-Zavala MG, Hernández-González SO, Jacques-Camarena O, Rodríguez-Morán M (2010). The product of triglycerides and glucose, a simple measure of insulin sensitivity. Comparison with the euglycemic-hyperinsulinemic clamp. J Clin Endocrinol Metab.

[24] Sanchez-Garcia A, Rodriguez-Gutierrez R, Mancillas-Adame L, Gonzalez-Nava V, Diaz Gonzalez-Colmenero A, Solis RC, Alvarez-Villalobos NA, Gonzalez-Gonzalez JG (2020). Diagnostic accuracy of the triglyceride and glucose index for insulin resistance: a systematic review. Int J Endocrinol.

[25] Khan SH, Sobia F, Niazi NK, Manzoor SM, Fazal N, Ahmad F (2018). Metabolic clustering of risk factors: evaluation of triglyceride-glucose index (TyG index) for evaluation of insulin resistance. Diabetol Metab Syndr.

[26] Sanchez-Inigo L, Navarro-Gonzalez D, Fernandez-Montero A, Pastrana-Delgado J, Martinez JA (2016). The TyG index may predict the development of cardiovascular events. Eur J Clin Invest.

[27] Luo E, Wang D, Yan G, Qiao Y, Liu B, Hou J, Tang C (2019). High triglyceride-glucose index is associated with poor prognosis in patients with acute ST-elevation myocardial infarction after percutaneous coronary intervention. Cardiovasc Diabetol.

[28] Guo Q, Feng X, Zhang B, Zhai G, Yang J, Liu Y, Liu Y, Shi D, Zhou Y (2022). Influence of the triglyceride-glucose index on adverse cardiovascular and cerebrovascular events in prediabetic patients with acute coronary syndrome. Front Endocrinol.

[29] Yu Q, Gao F, Ma XL (2011). Insulin says NO to cardiovascular disease. Cardiovasc Res.

[30] Zou X, Chen M, Sun L, Tan Q (2023). Hyperinsulinemia impaired coronary collateral circulation in patients with chronic total coronary occlusion. Diabetes Metab Syndr Obes.

[31] Muniyappa R, Montagnani M, Koh KK, Quon MJ (2007). Cardiovascular actions of insulin. Endocr Rev.

[32] He Z, Opland DM, Way KJ, Ueki K, Bodyak N, Kang PM, Izumo S, Kulkarni RN, Wang B, Liao R (2006). Regulation of vascular endothelial growth factor expression and vascularization in the myocardium by insulin receptor and PI3K/Akt pathways in insulin resistance and ischemia. Arterioscler Thromb Vasc Biol.

[33] Yan LJ (2014). Pathogenesis of chronic hyperglycemia: from reductive stress to oxidative stress. J Diabetes Res.

[34] Di Carli MF, Janisse J, Grunberger G, Ager J (2003). Role of chronic hyperglycemia in the pathogenesis of coronary microvascular dysfunction in diabetes. J Am Coll Cardiol.

[35] Demirbag R, Gur M, Yilmaz R, Kunt AS, Erel O, Andac MH (2007). Influence of oxidative stress on the development of collateral circulation in total coronary occlusions. Int J Cardiol.

[36] Glass CK, Olefsky JM (2012). Inflammation and lipid signaling in the etiology of insulin resistance. Cell Metab.

[37] Seiler CJE (2010). The human coronary collateral circulation. Eur J Clin Investig.

[38] Festa A, D'Agostino R, Hanley AJ, Karter AJ, Saad MF, Haffner SM (2004). Differences in insulin resistance in nondiabetic subjects with isolated impaired glucose tolerance or isolated impaired fasting glucose. Diabetes.

[39] Goldstein BJ (2002). Insulin resistance as the core defect in type 2 diabetes mellitus. Am J Cardiol.

[40] Petersen MC, Shulman GI (2018). Mechanisms of insulin action and insulin resistance. Physiol Rev.

