# Supplementary material for: Correlation between triglyceride glucose index and collateral circulation formation in patients with chronic total occlusion of coronary arteries in different glucose metabolic states
Source: Cardiovasc Diabetol. 2024 Jan 13;23:26. doi: 10.1186/s12933-023-02080-3 (PMC10787450; doi:10.1186/s12933-023-02080-3)
Supplement: Supplementary file 1 — Additional file 1: Table S1. Clinical baseline information according to the grouping of collateral circulation. Table S2. Poor collateral circulation formation in relation to various risk factors. Table S3. Comparison of TyG index and other factors AUC in different glucose metabolic states. Table S4. Comparison of AUC of TyG index in different glucose metabolic states. Figure S1. Biochemical indexes in different glucose metabolic states. a HDL-C, b TG, c TyG index, d FPG, e HbA1c. Figure S2. TyG index and poor CCC restricted cubic spline curves in different glucose metabolic states. a Normal glucose regulation, b prediabetes mellitus, c diabetes mellitus. Figure S3. TyG index and other factors predicted poor collateral circulation formation in different glucose metabolic states. [file 12933_2023_2080_MOESM1_ESM.docx]

**Table S1.** Clinical baseline information according to the grouping of collateral circulation

|  | **Poor CCC**  **(n=205)** | **Good CCC**  **(n=476)** | ***P*** |
| --- | --- | --- | --- |
| Age (years) | 60.19±10.15 | 59.58±10.00 | 0.773 |
| Male (n, %) | 154(75.1%) | 376(79.0%) | 0.129 |
| SBP (mmHg) | 133.33±17.43 | 133.54±17.73 | 0.735 |
| DBP (mmHg) | 78.84±11.24 | 78.55±1.34 | 0.960 |
| Smoking history (n, %) | 82(40.0%) | 203(42.6%) | 0.521 |
| Drinking history (n, %) | 55(26.8%) | 120(25.2%) | 0.657 |
| History of hypertension (n, %) | 121(59.0%) | 281(59.0%) | 0.998 |
| Previous medication |  |  |  |
| Antihypertensive drugs (n, %) | 118(57.6%) | 275(57.8%) | 0.959 |
| Lipid-lowering drugs (n, %) | 198(96.6%) | 450(94.5%) | 0.254 |
| Antiplatelet drugs (n, %) | 194(94.6%) | 441(92.6%) | 0.343 |
| antidiabetic drugs (n, %) | 78(38.0%) | 124(26.1%) | 0.002^*^ |
| Laboratory examination |  |  |  |
| cTNI(ng/mL) | 0.01(0.01-0.02) | 0.01(0.01-0.02) | 0.982 |
| NT-proBNP (pg/mL) | 123.50(81.65-168.20) | 126.00(79.71-174.00) | 0.654 |
| Cr (μmol/L) | 66.91±14.13 | 69.90±14.33 | 0.723 |
| eGFR (ml/min/1.73 m^2^) | 94.60 ±13.23 | 92.63±12.82 | 0.826 |
| CRP (mg/L) | 1.33(0.77-1.83) | 1.31(0.77-1.90) | 0.507 |
| TC (mg/dL) | 3.71(3.16-4.38) | 3.42(2.92-4.08) | ＜0.001^*^ |
| TG (mg/dL) | 1.85(1.27-2.60) | 1.32(0.98-1.71) | ＜0.001^*^ |
| HDL-C (mg/dL) | 0.90 (0.78-1.02) | 0.93(0.80-1.08) | 0.026^*^ |
| LDL-C((mg/dL) | 2.09(1.65-2.66) | 2.03(1.61-2.56) | 0.142 |
| FPG (mmol/L) | 7.24(5.31-9.43) | 5.17(4.60-6.28) | ＜0.001^*^ |
| HbA1c (%) | 7.60(6.10-9.20) | 6.10(5.70-6.90) | ＜0.001^*^ |
| LVEF (%) | 61.00(52.00-64.00) | 61.00(53.00-63.50) | 0.989 |
| TyG index | 9.35(8.81-9.70) | 8.67(8.31-8.99) | ＜0.001^*^ |
| Glucose metabolism status |  |  |  |
| NGR | 24(11.7%) | 115(24.2%) | ＜0.001^*^ |
| Pre-DM | 42(20.5%) | 176(37.0%) |  |
| DM | 139(67.8%) | 185(38.9%) |  |
| Number of vascular stenosis |  |  |  |
| 1 | 16(7.8%) | 46(9.7%) | 0.061 |
| 2 | 62(30.2%) | 104(21.8%) |  |
| 3 | 127(62.0%) | 326(68.5%) |  |
| CTO related artery |  |  |  |
| LAD | 85(35.1%) | 212(35.2%) | 0.414 |
| LCX | 67(27.7%) | 143(23.7%) |  |
| RCA | 90(37.2%) | 248(41.1%) |  |
| Rentrop collateral grading |  |  |  |
| 0 | 54(26.3%) |  |  |
| 1 | 151(73.7%) |  |  |
| 2 |  | 254(53.4%) |  |
| 3 |  | 222(46.6%) |  |

SBP Systolic blood pressure, DBP Diastolic blood pressure, cTnI: cardiac troponin I, NT-pro BNP: N-terminal B-type natriuretic peptide, Cr: creatinine, eGFR: estimated glomerular filtration rate, CRP: C-reactive protein, TC: total cholesterol, TG: triglyceride, HDL-C: high-density lipoprotein cholesterol, LDL-C: low-density lipoprotein cholesterol, FPG: fasting plasma glucose, HbA1c: glycated hemoglobin LVEF: left ventricular ejection fraction, TyG: triglyceride glucose, NGR: normal glucose regulation, Pre-DM: prediabetes mellitus, DM: diabetes mellitus, LAD: Left anterior descending artery, LCX: Left circumflex coronary artery, RCA: Right coronary artery, *: statistically significant difference between two groups

**Table S2.** Poor collateral circulation formation in relation to various risk factors

|  | **Univariate analysis** | | | **Multivariate analysis** | | |
| --- | --- | --- | --- | --- | --- | --- |
|  | **OR (95%Cl)** | **β** | ***P*** | **OR (95%Cl)** | **β** | ***P*** |
| TC | 1.403(1.177-1.673) | 0.339 | ＜0.001 | 1.089(0.874-1.356) | 0.085 | 0.448 |
| HDL-C | 0.349(0.163-0.748) | -1.053 | 0.007 | 1.155(0.436-3.059) | 0.144 | 0.773 |
| HbA1c | 1.679(1.497-1.883) | 0.518 | ＜0.001 | 1.278(1.120-1.458) | 0.245 | ＜0.001 |
| TyG index | 7.188(4.998-10.338) | 1.972 | ＜0.001 | 5.104(3.323-7.839) | 1.630 | ＜0.001 |

TC: total cholesterol, HDL-C: high-density lipoprotein cholesterol, HbA1c: glycosylated hemoglobin, TyG: triglyceride glucose, OR:Odds ratios CI: confidence interval

TC was adjusted for HDL-C、HbA1c、TyG index, HDL was adjusted for TC、HbA1c、TyG index, TyG index was adjusted for TC、HDL-C、HbA1c.

**Table S3. Comparison of TyG index and other factors AUC in different glucose metabolic states**

|  | Pair comparison of ROC curves | Difference between areas | *P* value |
| --- | --- | --- | --- |
| Total | TyG index-TC | 0.185 | ＜0.001 |
|  | TyG index-TG | 0.088 | ＜0.001 |
|  | TyG index-FPG | 0.048 | 0.027 |
|  | TyG index-HbA1c | 0.078 | 0.004 |
|  | TyG index-HDL-C | 0.208 | ＜0.001 |
| NGR | TyG index-TC | 0.040 | 0.683 |
|  | TyG index-TG | 0.017 | 0.364 |
|  | TyG index-FPG | 0.031 | 0.701 |
|  | TyG index-HbA1c | 0.001 | 0.993 |
|  | TyG index-HDL-C | 0.052 | 0.411 |
| Pre-DM | TyG index-TC | 0.020 | 0.001 |
|  | TyG index-TG | 0.122 | 0.032 |
|  | TyG index-FPG | 0.205 | 0.003 |
|  | TyG index-HbA1c | 0.197 | 0.006 |
|  | TyG index-HDL-C | 0.132 | 0.025 |
| DM | TyG index-TC | 0.035 | ＜0.001 |
|  | TyG index-TG | 0.099 | ＜0.001 |
|  | TyG index-FPG | 0.031 | 0.043 |
|  | TyG index-HbA1c | 0.081 | 0.012 |
|  | TyG index-HDL-C | 0.241 | ＜0.001 |

TC: total cholesterol, HDL-C: high-density lipoprotein cholesterol, HbA1c: glycosylated hemoglobin, TyG: triglyceride glucose, FPG: fasting plasma glucose.

**Table S4.** Comparison of AUC of TyG index in different glucose metabolic states

| Pair comparison of ROC curves | Difference between areas | *P* value |
| --- | --- | --- |
| NGR-Pre-DM | 0.165 | *P*=0.043 |
| NGR-DM | 0.236 | *P*＜0.001 |
| NGR-Total | 0.214 | *P=*0.001 |
| Pre-DM-DM | 0.071 | *P*=0.205 |
| Pre-DM-Total | 0.049 | *P*=0.367 |
| DM-Total | 0.022 | *P*=0.505 |

NGR: normal glucose regulation, Pre-DM: prediabetes mellitus, DM: diabetes mellitu.

**Figure S1** Biochemical indexes in different glucose metabolic states. **a** HDL-C, **b** TG, **c** TyG index, **d** FPG, **e** HbA1c.

| 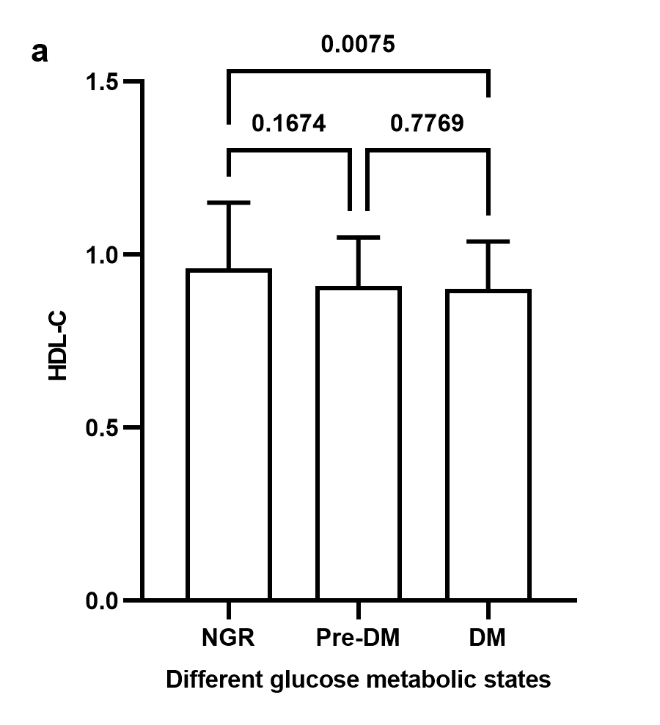 | 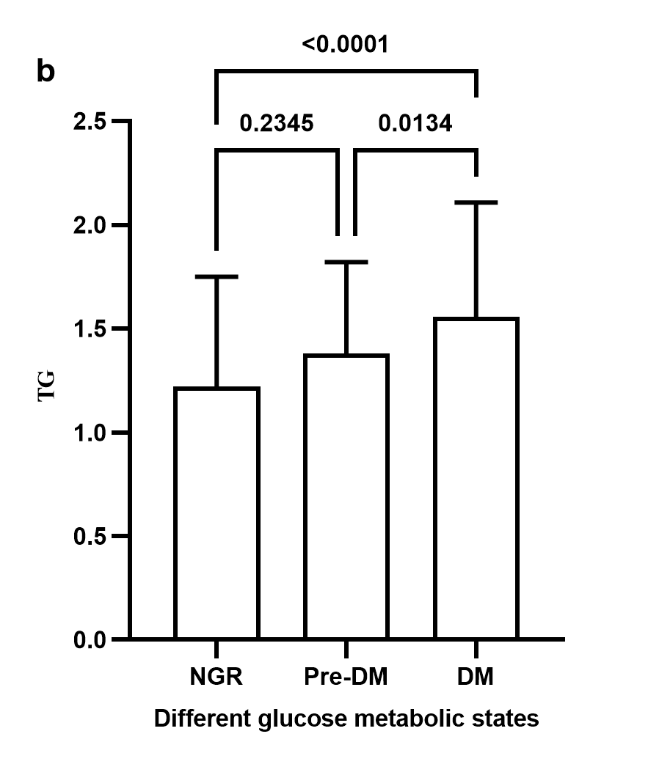 |
| --- | --- |
| 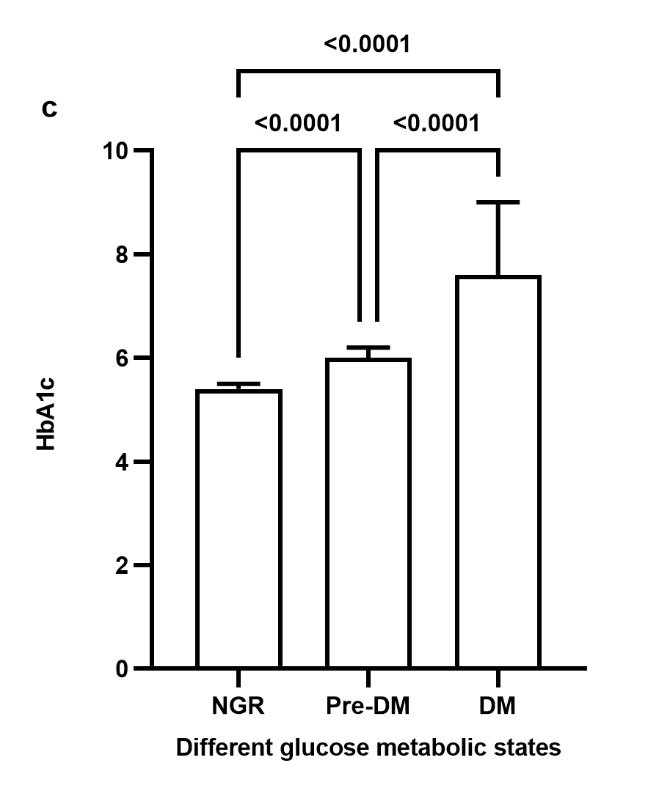 | 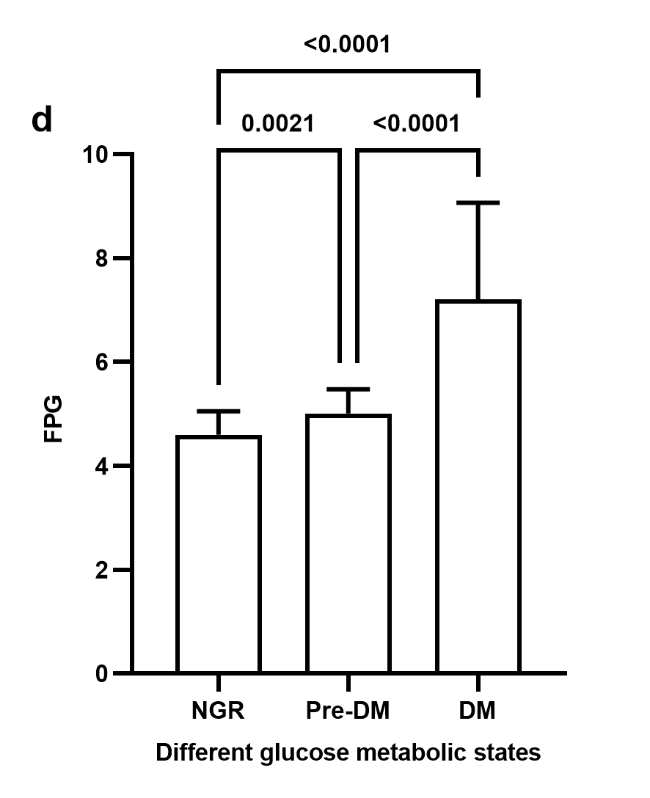 |
| 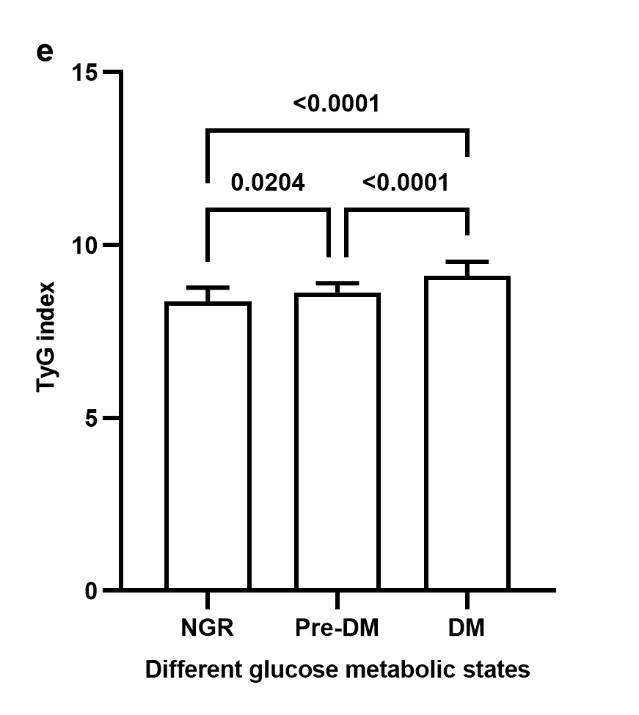 | |

**Figure S2** TyG index and poor CCC restricted cubic spline curves in different glucose metabolic states. a: normal glucose regulation, b: prediabetes mellitus, c: diabetes mellitu.

| **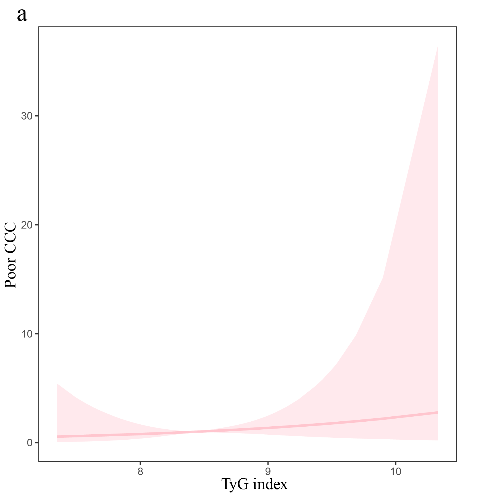** | **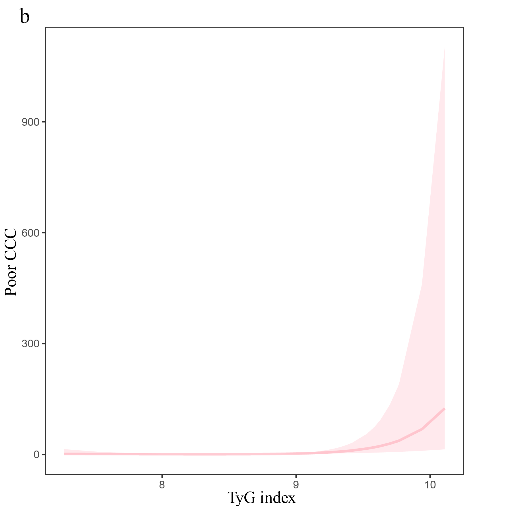** | **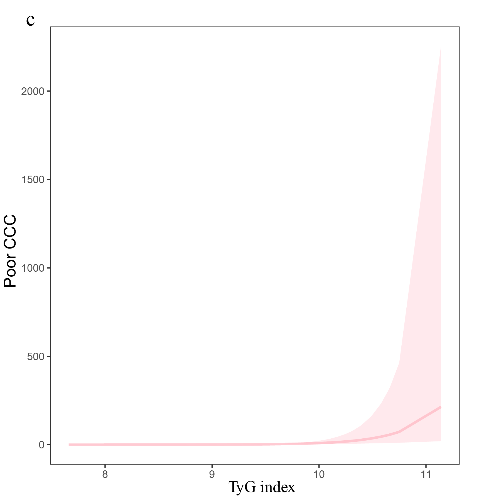** |
| --- | --- | --- |

**Figure S3. TyG index and other factors predicted poor collateral circulation formation in different glucose metabolic states.**

NGR: normal glucose regulation, Pre-DM: prediabetes mellitus, DM: diabetes mellitu.

| 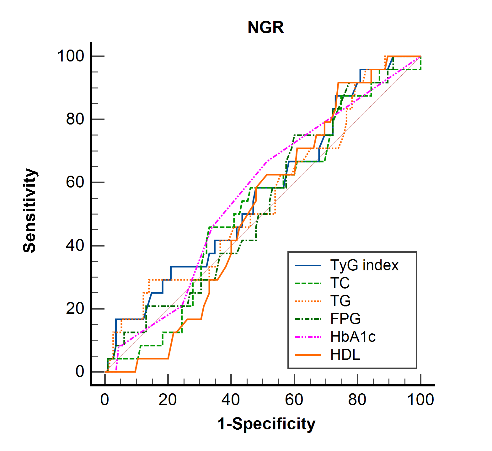 | 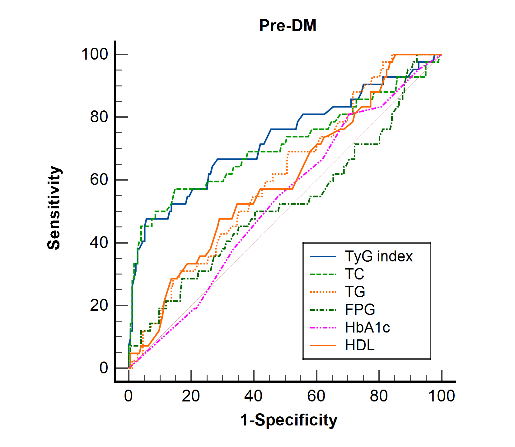 |
| --- | --- |
| 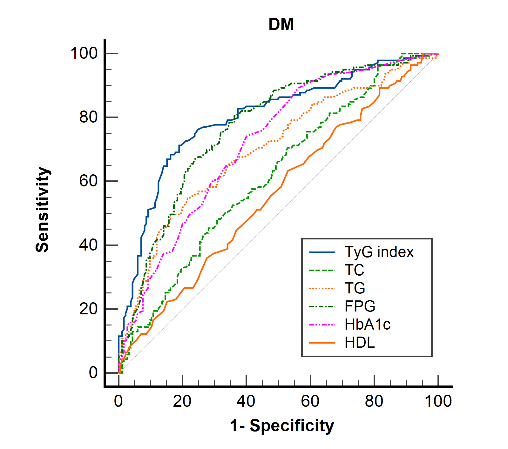 | 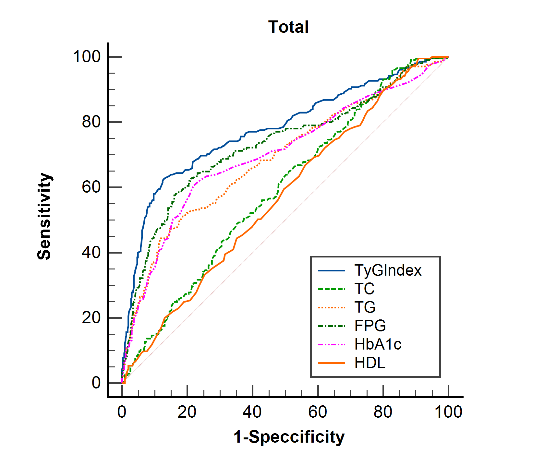 |
